# Severe Breakthrough COVID-19 Cases during Six Months of Delta Variant (B.1.617.2) Domination in Poland

**DOI:** 10.3390/vaccines10040557

**Published:** 2022-04-04

**Authors:** Piotr Rzymski, Monika Pazgan-Simon, Juliusz Kamerys, Anna Moniuszko-Malinowska, Katarzyna Sikorska, Joanna Wernik, Dorota Zarębska-Michaluk, Łukasz Supronowicz, Barbara Sobala-Szczygieł, Agata Skrzat-Klapaczyńska, Krzysztof Simon, Anna Piekarska, Piotr Czupryna, Małgorzata Pawłowska, Michał Brzdęk, Jerzy Jaroszewicz, Justyna Kowalska, Marcin Renke, Robert Flisiak

**Affiliations:** 1 Department of Environmental Medicine, Poznan University of Medical Sciences, 60-806 Poznań, Poland; 2 Integrated Science Association (ISA), Universal Scientific Education and Research Network (USERN), 60-806 Poznań, Poland; 3 1st Infectious Diseases Ward, Gromkowski Regional Specialist Hospital, 50-149 Wroclaw, Poland; monikapazgansimon@gmail.com; 4 Department of Infectious Diseases and Hepatology, Wrocław Medical University, 51-149 Wrocław, Poland; krzysimon@gmail.com; 5 Department of Infectious Diseases and Hepatology, Medical University of Łódź, 90-549 Łódź, Poland; juliuszkamerys@gmail.com (J.K.); annapiekar@gmail.com (A.P.); 6 Department of Infectious Diseases and Neuroinfections, Medical University of Białystok, 15-089 Białystok, Poland; annamoniuszko@op.pl (A.M.-M.); avalon-5@wp.pl (P.C.); 7 Department of Tropical Medicine and Epidemiology, Medical University of Gdańsk, 80-210 Gdańsk, Poland; ksikorska@gumed.edu.pl; 8 Department of Infectious Diseases and Hepatology, Faculty of Medicine, Collegium Medicum in Bydgoszcz, Nicolaus Copernicus University, 87-100 Toruń, Poland; joanna.wernik@gmail.com (J.W.); mpawlowska@cm.umk.pl (M.P.); 9 Department of Infectious Diseases, Jan Kochanowski University, 25-369 Kielce, Poland; dorota1010@tlen.pl (D.Z.-M.); michal.brzdek@gmail.com (M.B.); 10 Department of Infectious Diseases and Hepatology, Medical University of Białystok, 15-089 Białystok, Poland; l.supronowicz@wp.pl (Ł.S.); robert.flisiak1@gmail.com (R.F.); 11 Department of Infectious Diseases and Hepatology, Medical University of Silesia, 40-055 Katowice, Poland; sobala.szczygiel@op.pl (B.S.-S.); jerzy.jr@gmail.com (J.J.); 12 Department of Adults’ Infectious Diseases, Hospital for Infectious Diseases, Medical University of Warsaw, 02-091 Warsaw, Poland; agata.skrzatasw@gmail.com (A.S.-K.); jdkowalska@gmail.com (J.K.); 13 Division of Occupational, Metabolic and Internal Diseases, Institute of Maritime and Tropical Medicine, Faculty of Health Sciences, Medical University of Gdansk, 81-519 Gdynia, Poland; marcin.renke@gumed.edu.pl

**Keywords:** breakthrough infections, COVID-19 vaccines, SARS-CoV-2, booster dose, pandemic, inflammation, clinical outcome

## Abstract

The emergence of a highly transmissible and a more pathogenic B.1.617.2 (delta) variant of SARS-CoV-2 has brought concern over COVID-19 vaccine efficacy and the increased risk of severe breakthrough infections. The objective of this study was to assess the frequency and the clinical characteristics of severe breakthrough COVID-19 cases recorded in 10 Polish healthcare units between 1 June and 31 December 2021, a period during which a rapid surge in the share of B.1.617.2 infections was seen, while a significant number of populations were already fully vaccinated. Overall, 723 individuals who completed the initial vaccination regime (fully vaccinated group) and an additional 18 who received a booster dose were identified—together, they represented 20.8% of all the COVID-19 patients hospitalized during the same period in the same healthcare institutions (0.5% in the case of a group that received a booster dose). Although laboratory and clinical parameters did not differ between both groups, patients who received a booster tended to have lower CRP, IL-6, PCT, and d-dimer levels and they required oxygen therapy less frequently. The most common early COVID-19 symptoms in the studied group were fatigue, cough, fever (>38 °C), and dyspnea. Individuals with no detectable anti-spike IgG antibodies constituted 13%; the odds of being a humoral non-responder to the vaccine were increased in patients aged >70 years. Fully vaccinated patients hospitalized after more than 180 days from the last vaccine dose were significantly older and they were predominantly represented by individuals over 70 years and with comorbidities, particularly cardiovascular disease. Contrary to mRNA vaccines, most patients vaccinated with adenoviral vector vaccines were infected within six months. A total of 102 fatal cases (14% of all deaths among vaccinated individuals; 0.7% in the case of a group that received a booster dose) were recorded, representing 17.6% of all the COVID-19 fatalities recorded in June–December 2021 in the considered healthcare units. The odds of death were significantly increased in men, individuals aged >70 years, patients with comorbidities, and those identified as humoral non-responders to vaccination; in fully vaccinated patients the odds were also increased when the second vaccine dose was given >180 days before the first COVID-19 symptoms. The mortality rate in immunocompromised subjects was 19%. The results indicate that compared to vaccinated individuals, severe COVID-19 and deaths in the unvaccinated group were significantly more prevalent during the B.1.617.2-dominated wave in Poland; and, it highlight the protective role of a booster dose, particularly for more vulnerable individuals.

## 1. Introduction

The clinical trials and post-authorization studies have shown that COVID-19 vaccines are highly effective in preventing severe disease [1]. In the United States alone, vaccination was estimated to prevent over 10 million hospitalizations and 1 million COVID-19-related deaths by November 2021 [2]. Although the protection against an infection decreased over time due to the emergence of more transmissible SARS-CoV-2 variants with abilities to partially evade humoral immunity and to induce higher viral loads in the upper respiratory tract, as well as due to the gradual waning of neutralizing antibodies a few months after vaccination [3,4], the adaptive cellular immunity, pivotal for an effective antiviral response, mainly remained intact and robust [5,6]. Furthermore, the initial and the considerable levels of protection against infection could be restored with a booster dose, which became available in various world regions for individuals who finished the primary vaccination regime at least six months ago [7,8]. Despite it, some vaccinated individuals may still experience severe or critical COVID-19 due to a poorer response to immunization. This may result from primary and secondary immune deficiencies, immunosenescence in the elderly, and various lifestyle factors [9,10,11].

Our previous investigation [12] showed that in the first five months of the vaccination campaign in Poland, severe breakthrough cases of COVID-19 constituted a minute fraction of all COVID-19 patients requiring hospitalizations, and they were primarily represented by partially vaccinated individuals. The fully vaccinated (at least 14 days after the second dose) group comprised only 0.15% of all COVID-19 hospitalizations in the monitored clinical units, and it was frequently represented by immunocompromised patients, including vaccine non-responders identified by the negative results of the anti-SARS-CoV-2 spike protein IgG serological test [12]. However, the cut-off date for this analysis was 31 May 2021, and it preceded a period of B.1.617.2 (delta) variant domination in Poland. In turn, long-term studies have suggested that the pathogenicity of SARS-CoV-2 was undergoing various changes throughout the pandemic, with an increasing frequency of respiratory symptoms and higher levels of inflammatory markers in hospitalized patients [13]. Animal studies indicated that B.1.617.2 revealed significantly enhanced pathogenicity linked to P618R mutation in spike protein [14], while epidemiological observations evidenced an increased hospital admission and emergency care attendance risk for patients infected with this variant [15]. Whether severe COVID-19 cases in vaccinated individuals infected with B.1.617.2 displayed any distinctive clinical features was not a subject of the study.

Therefore, the objective of the present study was to assess the frequency and the clinical characteristics of severe breakthrough COVID-19 cases recorded in 10 Polish healthcare units between 1 June and 31 December 2021, a period during which a rapid surge in the share of B.1.617.2 infections was seen, while a significant number of population was already fully vaccinated. To this end, symptomatology, levels of inflammation markers, hematological parameters, and the overall clinical course of disease were analyzed in the hospitalized patients who completed the initial COVID-19 vaccine protocol and those who received the booster dose.

## 2. Materials and Methods

### 2.1. Study Design 

The clinical and the demographical data on patients who received at least 1 dose of the COVID-19 vaccine and were later hospitalized due to COVID-19 between 1 June 2021 and 31 December 2021 in 10 Polish healthcare units located in cities (2 units in Białystok, and 1 in Bydgoszcz, Bytom, Gdańsk, Katowice, Kielce, Łódź, Warsaw and Wrocław) were extracted and retrospectively analyzed. All of these units were located in the University Hospitals, so they were leading departments in particular regions of Poland. Between June and December 2021, the number of newly identified SARS-CoV-2 infections in Poland increased by 1.24 million, while the share of fully vaccinated individuals in the Polish population reached 55.5% [16]. Four COVID-19 vaccines were in use: BNT162b2 (BioNTech/Pfizer, Mainz, Germany/New York, NY, USA), mRNA-1273 (Moderna Therapeutics, Cambridge, MA, USA), AZD-1222 (Oxford/AstraZeneca, UK/Sweden), and Ad26.COV2.S (Janssen/Johnson&Johnson, Beerse, Belgium/New Brunswick, NJ, USA). Since September 2021, the booster dose of mRNA vaccines became available for those who completed an initial vaccine regime at least six months ago. By the end of the period considered in this study, 18.1% of the Polish population received a booster [16]. The total monthly share of B.1.617.2 infections in Poland was as follows: June—17.8%, July—77.6%, August—75.4%, September—94.4% and October—99.9%, November—99.9%, and December—95.8% [17].

The study considered COVID-19 patients who were fully vaccinated (received the second dose of BNT162b, mRNA-1273, or AZD1222—or a single dose of Ad26.COV.S vaccine at least 14 days before the onset of the first COVID-19 symptoms) as well as patients who received a booster dose at least 14 days before the onset of first symptoms.

This study had a retrospective and a non-interventional nature, and it was based on data collected in the national SARSTer database. Therefore, it did not require approval of the Bioethics Committee or written consent. The patients’ data was protected according to the European Union General Data Protection Regulation.

### 2.2. Data Extraction

The demographical data included age, gender, BMI, and comorbidities. Based on the latter and on reported chronic use of medicines, individuals with immunosuppression were identified. Laboratory parameters at admission included C-reactive protein (CRP), d-dimer, interleukin-6 (IL-6), procalcitonin (PCT), white blood cell count (WBC), absolute lymphocyte count (ALC), absolute neutrophil count (ANC), and platelet count (PLC). The neutrophil-to-lymphocyte ratio (NLR), the prognostic marker of severity and outcome, was calculated. The following values were used to define a hyperinflammatory state: CRP > 200 mg/L, IL-6 > 100 pg/mL, PCT > 0.25 ng/mL and WBC > 11 × 10^3^/µL [18]. Early symptoms of infection before the treatment (cough, dyspnea, fever, headache, fatigue, nausea, diarrhea, vomiting, and anosmia) were recorded. Oxygen saturation (SpO_2_) and lung involvement based on computed tomography (CT) imaging were recorded upon admission. Patients were defined as humoral non-responders to a vaccine if they had a negative result of the serological test (anti-SARS-CoV-2 spike protein IgG antibodies) at admission. The clinical state of patients was assessed during admission with the ordinal scale based on WHO recommendations but modified to an 8-score version to fit the specificity of the national health care system, as in previous Polish SARSTer project investigations [18,19]. The scores were defined as follows: (1) not hospitalized, no activity restrictions; (2) not hospitalized, no activity restrictions and/or requirement of oxygen supplementation at home; (3) hospitalized, not requiring oxygen supplementation or medical care; (4) hospitalized, requiring no oxygen supplementation but requiring medical care; (5) hospitalized, requiring normal oxygen supplementation; (6) hospitalized, on non-invasive ventilation with high-flow oxygen equipment; (7) hospitalized for invasive mechanical ventilation or extracorporeal membrane oxygenation; and (8) death. Patients were diagnosed and treated according to the updated national recommendations for COVID-19 management [19,20].

### 2.3. Statistical Analysis

The data was analyzed with Statistica v. 13 (StatSoft, Tulsa, OK, USA). For continuous variables (age, BMI, time from administration of the last vaccine dose, length of hospitalization, and laboratory markers), differences between the fully vaccinated and the booster groups were tested with a Student’s *t*-test. Data expressed on the nominal scale (WHO classification) was evaluated with the Mann–Whitney U test. For nominal categorical variables, differences in frequencies were tested with Pearson’s χ2 test. The association between demographic characteristics (age > 70 years, BMI > 30 kg/m^2^, gender, comorbidities, immunosuppression, and vaccine non-responders) and death due to COVID-19 was evaluated with odds ratios (ORs) with a 95% confidence interval (CI) calculated according to the formulas given by Bland and Altman using MedCalc (MedCalc, Ostend, Belgium). A *p*-value < 0.05 was considered statistically significant.

## 3. Results

### 3.1. Demographic Characteristics

A total of 741 COVID-19 patients hospitalized in the considered Polish healthcare units between June and December 2021 were considered in this study. Among them, 97.6% (*n* = 723) individuals completed an initial vaccination regime at least 14 days before the onset of the first disease symptoms, while 2.4% (18) patients had received a booster dose before hospitalization. The fully vaccinated patients and those who received a booster constituted, respectively, 20.3% and 0.5% of all the COVID-19 patients hospitalized during the same period in the same healthcare institutions.

Most of the hospitalized individuals suffered from at least one chronic disease (predominantly represented by cardiovascular diseases), while half were aged >70 years, and approximately one quarter were obese (Table 1). Overall, 11.6% of patients had a primary or secondary immune deficiency, while 12.9% met the criteria of vaccine non-responders. Nearly half of the patients in the fully vaccinated group were hospitalized after more than 180 days (6 months) from receiving the last vaccine dose. The majority of these individuals (86.8%) had detectable levels of the anti-SARS-CoV-2 spike protein IgG antibodies status at admission.

The age of vaccine non-responders ranged from 26 to 93 years (mean ± SD 69.2 ± 17.0). Age > 70 years significantly increased odds of being non-responder (OR, 95% CI: 1.6, 1.1–2.6; *p* < 0.05), contrary to other factors (obesity, gender, comorbidities, and immunosuppression). Only 11.5% (*n* = 11) of vaccine non-responders were immunocompromised.

### 3.2. Clinical Characteristics

**Figure 1:**
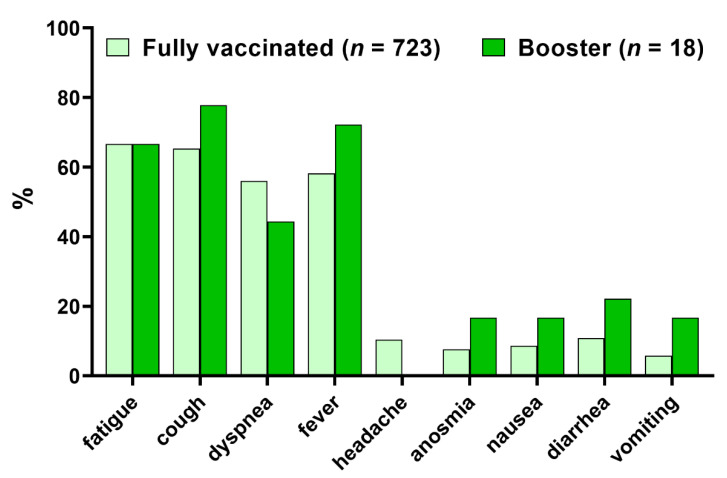
Frequency of early COVID-19 symptoms in vaccinated individuals requiring hospitalization (*n* = 741).

The most common early symptoms of COVID-19 in the studied group were fatigue (66.7%), cough (65.6%), fever (>38 °C) (59.8%), and dyspnea (55.7%) (Figure 1). No significant difference between the fully vaccinated and the booster groups was found. The laboratory and the clinical characteristics of the studied patients are summarized in Table 2. Baseline severity did not differ between groups, with most patients (55.8%) hospitalized, requiring medical care but not oxygen supplementation. Inflammatory markers (CRP, IL-6, PCT, and WBC) did not exceed thresholds for the hyperinflammatory state. Although laboratory and clinical parameters did not differ between both groups, patients who received a booster tended to have lower CRP, IL-6, PCT, and d-dimer levels and require oxygen therapy less frequently (Table 2).

Overall, 102 patients (13.8%) had a fatal outcome, among which 98 deaths occurred in the fully vaccinated group (13.6%), while 4 were in the booster group (22.2%). Fatal cases in the fully vaccinated patients and those who received a booster constituted, respectively, 17.0% and 0.5% of all deaths due to COVID-19 during the same period in the same healthcare institutions.

Fatal cases of the vaccinated represented 17.6% of all deaths due to COVID-19, observed between June and December 2021 in healthcare units considered in the study. The demographic, laboratory, and clinical characteristics of fatal cases are given in Table 3.

**Figure 2:**
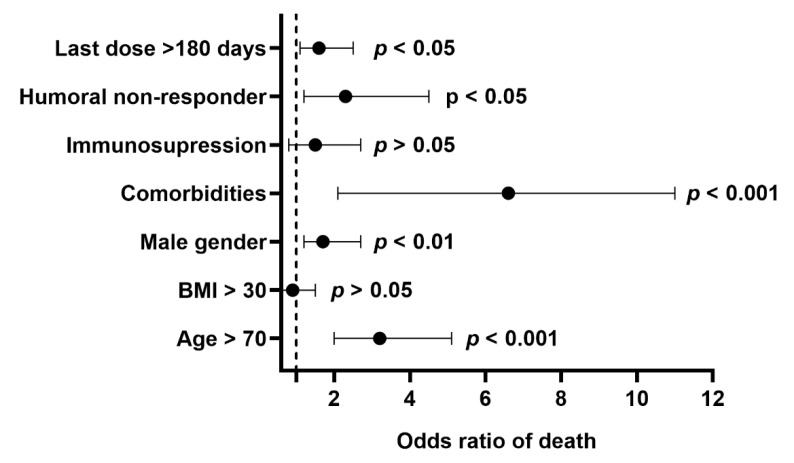
The odds (95% confidence interval) of death in vaccinated individuals requiring hospitalization related to various patient characteristics (*n* = 741). The last dose > 180 days parameter was assessed for fully vaccinated individuals (*n* = 723).

Compared to survivors, patients who died had higher lung involvement (25.9 ± 20.1 vs. 41.6 ± 25.9%, *p* < 0.001), lower SpO_2_ at admission (91.1 ± 5.8 vs. 84.0 ± 13.6, *p* < 0.001), increased WBC (8.0 ± 8.6 vs. 10.3 ± 11.7, *p* < 0.05) and NLR (6.5 ± 6.3 vs. 22.6 ± 9.5, *p* < 0.001), but lower PTC (214.3 ± 98.5 vs. 189.9 ± 98.1, *p* < 0.001), elevated concentrations of CRP (78.9 ± 76.8 vs. 127.4 ± 91.8, *p* < 0.001), IL-6 (81.5 ± 286.8 vs. 274.7± 604.5, *p* < 0.001), and d-dimer (1655 ± 4835 vs. 4257 ± 14609, *p* < 0.001). Being a man, a vaccine non-responder, and more than 70 years old or possessing at least one comorbidity increased the odds of death due to COVID-19; in fully vaccinated individuals, the odds were also increased when the last vaccine dose was given more than 180 days prior to the first COVID-19 symptoms (Figure 2). The mortality rate was 18.6% for immunosuppressed patients and 26.0% among vaccine non-responders.

The majority of hospitalized patients who received a single dose of Ad26.COV2.S vaccine (85.1%) presented the first COVID-19 symptoms before 180 days from their administration. In the case of patients vaccinated with AZD1222, 77.9% received the second dose earlier within 180 days prior to the first symptoms. In comparison, most hospitalized patients vaccinated with mRNA-1273 or BNT162b2 presented the first disease symptoms after at least 180 days from the second dose—41.2 and 37.6%, respectively. The detailed comparison of demographic and clinical characteristics of fully vaccinated patients who received the last dose more and less than 180 days before the onset of the first COVID-19 symptoms is presented in Table 4. As revealed, patients representing the former group were significantly older, suffered more frequently from cardiovascular disease, and had a higher level of d-dimer at admission. No other differences between these two groups were observed.

## 4. Discussion

This paper provides a clinical characteristic of vaccinated individuals hospitalized due to breakthrough infection during six months of emergence and domination of B.1.617.2 variant in Poland. The frequency of hospitalizations and deaths due to COVID-19 was nearly 5-fold and over 5.5-fold lower among the vaccinated than among other patients. This is a reassuring finding, indicating that COVID-19 vaccines retained their primary goal of decreasing the clinical severity of disease, including during the spread of a highly transmissible, better adapted to evade humoral immunity, and more pathogenic SARS-CoV-2 variant [7,21,22]. This is in line with the previous observation of a negative relationship between the vaccination status of particular European Economic Area populations and rates of hospitalizations, admissions to intensive care units, and deaths due to COVID-19 during the autumn SARS-CoV-2 wave in 2021 [23]. Altogether these results encourage the continued evidence-based promotion of COVID-19 vaccinations, using different channels to overcome vaccine hesitancy in regions such as Poland—especially in rural areas where the vaccination rate was lower than in the cities [24,25,26]. The decision to refuse the COVID-19 vaccination among Polish patients was most frequently associated with fears over side effects, a conviction that comorbidities excluded them from vaccination, and conspiracy beliefs; and, it was often undertaken under the influence of online information and the opinions of relatives and friends [27].

As found in the present study, half of the patients who completed the initial vaccine regime received the last vaccine dose more than 180 days (6 months) prior to the onset of the first COVID-19 symptoms. The longitudinal analyses found that humoral response substantially decreased six months after receiving the second dose of AZD-1222 or mRNA vaccines, especially among the elderly [28,29,30]. This is in line with our observations, according to which fully vaccinated patients hospitalized after more than 180 days from the last vaccine dose were significantly older and predominantly represented by individuals over 70 years. A single dose of Ad26.COV2.S elicited relatively stable antibody levels throughout the 8-month follow-up period, but their initial levels were significantly lower than that observed after two doses of other COVID-19 vaccines [31]. Notably, in the present study, most hospitalized patients who received a single dose of Ad26.COV2.S presented early disease symptoms before 180 days from vaccine administration. In the case of hospitalized patients previously vaccinated with one of the mRNA vaccines, the onset of the first symptoms was noted more than six months after administering the second dose. This agrees with comparative analyses of the effectiveness of different vaccine types in preventing COVID-19 hospitalizations in a real-world setting, which have shown that mRNA vaccines are superior to a single dose of Ad26.COV2.S. Furthermore, the present study demonstrated that over three quarters of hospitalized patients vaccinated with AZD1222 had received a second dose less than 180 days before developing COVID-19. It may indicate lower efficacy of this vaccine against hospitalization due to B.1.617.2 compared to mRNA vaccines, for which the majority of hospitalized patients had received the second dose more than six months prior to COVID-19. In line with this, a meta-analysis of clinical studies has shown decreased effectiveness of AZD1222 after the second dose against B.1.617.2 variant hospitalizations [32].

The gradual waning of the humoral immune response increases the risk of infection; additionally, it is magnified when novel viral variants can partially evade the neutralization of neutralizing antibodies. As shown in the large retrospective study, the initial high effectiveness of the BNT162b2 vaccine against infection decreased a few months after administering the second dose regardless of the SARS-CoV-2 variant, but the most profound decline was seen in the case of B.1.617.2 [33]. The comparative analysis conducted on a sample of US veterans has shown the protection offered by a single dose of Ad26.COV2.S to 13% after 6 months. The risk of breakthrough infection is not directly equal to the risk of severe disease requiring hospitalization as a pivotal antiviral function in vaccinated individuals is played by adaptive cellular immunity [34]. However, some vaccinated individuals may still be prone to acute infection. As demonstrated in the present study for the fully vaccinated group, receiving the last dose more than 180 days before the onset of the first COVID-19 symptoms significantly increased odds of death in hospitalized patients. All in all, this supports the use of a booster dose after six months in order to restore high antibody levels, increase their neutralization activities and provide additional protection against SARS-CoV-2 infection and severe illness [7,35,36].

No statistically significant difference in clinical parameters was found between individuals who completed the initial vaccine regime and those who received a booster, although the latter group’s size was small. However, some tendency for a lower CRP, IL-6, PCT, and d-dimer, and a less frequent requirement for oxygen supplementation were noted in patients who received a booster at least 14 days prior to their first COVID-19 symptoms. As demonstrated in other studies during the period dominated by the B.1.617.2 variant, the efficacy of a booster dose against hospitalization within three months of administration was very high, and it exceeded 90% [37,38]. Our data suggest that a booster dose may potentially benefit those who experience a more severe COVID-19, but this would require confirmation from a larger sample size. Nevertheless, the hospitalizations of those who received a booster dose was rare in Poland during the first 4 months during which its administration was officially recommended, and its uptake in the general population reached 18.1% by the end of December 2021) [37]. At the same time, more than one million newly detected infections, nearly entirely caused by B.1.617.2, were detected in the country [38]. These findings encourage the further promotion of booster doses in the population to decrease the disease burden. According to previous research conducted in September 2021, the majority (71%) of Poles who completed an initial vaccine regime declared a willingness to receive a booster dose [39].

Importantly, 12% of vaccinated patients considered in this study suffered from a primary or a secondary immune deficiency. These patients were also present among those who received a booster dose and those who died due to COVID-19. It highlights that some immune deficient subjects may remain vulnerable to severe COVID-19 and death despite repeated vaccine administration. Therefore, these patients need to continue to adhere to preventive measures, especially during the increased number of SARS-CoV-2 infections, which is expected for the temperate zone between autumn and spring [40]. Furthermore, pre-exposure prophylaxis based on the combination of monoclonal antibodies should be considered in vaccinated patients with immune deficiencies, particularly those who fail to generate a humoral response [41]. However, it should be stressed that selecting particular antibodies for such a purpose requires ensuring that they retain neutralization of all epidemiologically-relevant SARS-CoV-2 lineages. In turn, this became increasingly challenging due to the emergence of heavily mutated variants, such as selected omicron subvariants that evade the activity of various monoclonal antibodies, including tixagevimab/cilgavimab formulated for slow release to be used prophylactically primarily in immunocompromised individuals and otherwise effective against other important viral variants [42]. Additional vaccine doses in the future or development of the novel COVID-19 vaccines ensuring better immunogenicity in patients experiencing immunosuppression may be required to decrease the risk of severe disease in this group. One of the promising vaccine candidates in this regard includes those based on self-amplifying RNAs, which enhance antigen presentation and may subsequently mount a robust adaptive immune response [43].

Although some of the immunocompromised patients were identified as humoral non-responders to the vaccination, the latter group (represented by one quarter of hospitalized patients) predominantly included individuals without primary or secondary immunodeficiency (88%). As demonstrated, BMI, gender, or comorbidities were not associated with a lack of humoral response, contrary to age > 70 years. This is in line with previous serological surveys, indicating lower frequencies of neutralizing antibodies and a higher rate of no response to COVID-19 vaccination in the elderly group, sometimes with no apparent association with medication [44,45].

Study limitations should be stressed. This is a retrospective clinical analysis based on data from selected healthcare units in Poland. Although it clearly shows that hospitalizations and deaths due to COVID-19 were more frequent among unvaccinated individuals, caution should be made in extrapolating the results to the general situation in Poland during June–December 2021. The size of the group that received a booster dose was small. Although the study indicates that such individuals rarely required hospitalization, the comparison of their clinical characteristics with patients who only completed an initial vaccination protocol should be interpreted with caution, and further study will be required to draw more definitive conclusions. Moreover, no general conclusions should be drawn on the differences in the frequency of hospitalizations between patients vaccinated with particular vaccines as the rate of their administration in Poland was related to the local availability and occupational and age groups. The frequencies observed in the present study closely reflected the total distribution of particular vaccines in the country. In 2021, most Poles received BNT162b, followed by AZD1222, Ad26.COV2.S, and mRNA-1273 [45]. One should also note that, similar to various previous studies, the vaccine non-responders were identified in this study on the basis of the anti-SARS-CoV-2 spike protein IgG antibodies status [12,45,46,47]. However, a lack of neutralizing antibodies may not always reflect the complete lack of an adaptive response. As documented in the literature, some patients, particularly those with immunosuppression, may only generate a cellular response [48], evaluation of which was not possible in routine clinical settings. Last but not least, the study results cannot be extrapolated to the infection and the hospitalization wave caused by the B.1.1.529 (omicron) variant, which was identified in Africa in mid-November 2021 and rapidly spread to other regions, including Poland where its prevalence started to increase in January 2022 [17]. However, they may represent a good reference point for further studies conducted under the domination of omicron or future SARS-CoV-2 variants.

## 5. Conclusions

This paper documented the clinical course of COVID-19 in vaccinated patients who required hospitalization during the emergence and the domination of a highly transmissible, better adapted to evade humoral immunity, and more pathogenic B.1.617.2 variant. The results indicate that individuals who completed the initial vaccination regime constituted only one fifth of all hospitalizations recorded in the considered period and one sixth of all deaths. Moreover, they also highlight the protective role of a booster dose, given within six months from the initial vaccination regime, particularly in more vulnerable subjects. Such patients represented only a minute fraction of all hospitalizations and deaths recorded in the studied period and in the selected healthcare units. This study further reassures the high efficacy of the COVID-19 vaccines in preventing severe disease, and it provides a reference point for further studies conducted under the dominance of other SARS-CoV-2 variants.

## Figures and Tables

**Table 1:** The demographic characteristics of the studied group of vaccinated patients hospitalized with COVID-19.

Parameter	Fully Vaccinated	Booster Dose	*p*-Value
	(*n* = 723)	(*n* = 18)	
**Age**			
Mean ± SD, years	68.2 ± 15.8	73.9 ± 16.6	ns
Range, years	18–99	25–91	
>70 years, % (*n*)	49.5 (358)	72.2 (13)	ns
**Female/Male, % (*n*)**	43.9 (317)/56.1 (406)	33.3 (6)/66.7 (12)	ns
**BMI**			
Mean ± SD, kg/m^2^	28.6 ± 5.7	25.4 ± 3.0	<0.05
Range, kg/m^2^	15.6–54.9	19.5–31.0	
Obesity (≥30.0), % (*n*)	26.6 (192)	11.1 (2)	ns
**Comorbidities, % (*n*)**	84.9 (614)	94.4 (17)	ns
Asthma, % (*n*)	7.6 (55)	16.7 (3)	ns
Cardiovascular, % (*n*)	73.9 (534)	83.3 (15)	ns
Cancer, % (*n*)	12.7 (92)	16.7 (3)	ns
Chronic kidney disease, % (*n*)	18.3 (132)	33.3 (6)	ns
Diabetes, % (*n*)	30.4 (220)	38.9 (7)	ns
**Immunosuppression, % (*n*)**	11.1 (80)	33.3 (6)	<0.01
**Non-responders, % (*n*)**	13.0 (94)	11.1 (2)	ns
**Days from the last vaccine dose**			
Mean ± SD (range)	181.1 ± 56.3 (15–329)	56.3 ± 70.0 (15–321) *	-
>180 days, % (*n*)	50.2 (362)	-	

*—one patient received a booster dose in May 2021 before official recommendations for boosters were issued in November 2021. ns—no significant difference (*p* > 0.05).

**Table 2:** Laboratory parameters (mean ± SD) at admission and clinical characteristics of vaccinated patients hospitalized with COVID-19.

Parameter	Fully Vaccinated (*n* = 723)	Booster Dose (*n* = 18)	*p*-Value
**ALC** *, ×10^3^/µL, mean ± SD	1.2 ± 1.4 a	1.0 ± 0.7	ns
**ANC**, ×10^3^/µL, mean ± SD	6.0 ± 6.1	8.0 ± 7.4	ns
**NLR** *, mean ± SD	7.3 ± 7.2	8.4± 7.3	ns
**PLC**, ×10^3^/µL, mean ± SD	211.9 ± 99.4	171.7 ± 50.3	ns
**WBC**, ×10^3^/µL, mean ± SD	8.3 ± 9.2	8.9 ± 7.0	ns
>11 × 10^3^/µL, % (*n*)	14.6 (106)	11.1 (2)	ns
**CRP, mg/L, mean ± SD**	85.8 ± 81.2	75.7 ± 60.2	ns
>100 mg/L, % (*n*)	34.0 (246)	2.8 (5)	ns
**IL-6, pg/mL, mean ± SD**	109.3 ± 357.3	55.7 ± 69.5	ns
>100 pg/mL, % (*n*)	13.4 (97)	5.5 (1)	ns
**PCT, ng/mL, mean ± SD**	3.6 ± 16.9	0.1 ± 0.2	ns
>0.25 ng/mL, % (*n*)	23.2 (168)	11.1 (2)	ns
**Received vaccine**			-
BNT162b2, % (*n*)	64.0 (463)	100.0 (18)	
mRNA-1273, % (*n*)	7.1 (51)		
AZD1222, % (*n*)	18.8 (136)		
AD26.COV2.S, % (*n*)	9.4 (68)		
Heterologous, % (*n*)	0.7 (4)		
**d-dimer, ng/mL**, mean ± SD	2031.1 ± 7144.2	1193.6 ± 1492.9	ns
**Hospital stay**, days	11.8 ± 8.6	12.5 ± 7.4	ns
**Baseline WHO**, median, (IQR)	4 (4–5)	4 (4–4)	ns
**Admission SpO_2_**, %	90.0 ± 7.9	92 ± 4.3	ns
**Lung involvement**, %	27.7 ± 21.5	28.4 ± 22.8	ns
**Oxygen therapy**, % (*n*)	81.2 (587)	11.1 (2)	ns
**Mechanical ventilation**, % (*n*)	4.0 (29)	5.5 (1)	ns

ns—no significant difference (*p* > 0.05); ALC—absolute lymphocyte count, ANC—absolute neutrophil count, NLR—neutrophil-to-lymphocyte ratio, PLC—platelet count, WBC—white blood cells count, CRP—C-reactive protein, IL-6—interleukin-6, PCT—procalcitonin. *—leukemia patients (*n* = 7) were excluded from this statistic.

**Table 3:** The demographic, laboratory, and clinical characteristics of vaccinated COVID-19 patients with fatal outcomes (*n* = 102).

Parameter	Fully Vaccinated	Booster Dose	*p*-Value
	(*n* = 98)	(*n* = 4)	
**Age**			
Mean ± SD, years	78.7 ± 10.6	73.2 ± 13.6	ns
>70 years, % (*n*)	74.5 (73)	50.0 (2)	ns
**Female/Male**, % (*n*)	32.6 (32)/67.4 (66)	25.0 (1)/75.0 (3)	ns
**BMI**			
Mean ± SD, kg/m^2^	27.9 ± 6.3	25.5 ± 2.5	ns
Obesity (≥30.0), % (*n*)	19.4 (19)	0.0 (0)	ns
**Comorbidities**, % (*n*)	96.9 (95)	100 (4)	ns
Asthma, % (*n*)	5.1 (5)	25.0 (1)	ns
Cardiovascular, % (*n*)	87.7 (86)	75.0 (3)	ns
Cancer, % (*n*)	73.5 (72)	0.0 (0)	ns
Chronic kidney disease, % (*n*)	33.7 (33)	25.0 (1)	ns
Diabetes, % (*n*)	36.7 (36)	50.0 (2)	ns
**Immunosuppression**, % (*n*)	14.3 (14)	50.0 (2)	ns
**Humoral non-responders**, % (*n*)	16.3 (16)	25.0 (1)	ns
**Days from the last vaccine dose**			-
Mean ± SD (range)	194.4 ± 53.9 (72–303)	50.5 ± 27.3 (17–83)	
>180 days, % (*n*)	58.2 (57)	-	
**ALC** *, ×10^3^/µL, mean ± SD	1.0 ± 1.9	0.5 ± 0.8	ns
**ANC**, ×10^3^/µL, mean ± SD	8.7 ± 12.6	29.4 ± 42.3	<0.05
**NLR** *, mean ± SD	21.8 ± 95.6	56.2 ± 81.5	ns
**PLC**, ×10^3^/µL, mean ± SD	191.7 ± 99.4	144.8 ± 38.2	ns
**WBC**, ×10^3^/µL, mean ± SD	10.2 ± 11.8	11.4 ± 9.2	ns
>11 × 10^3^/µL, % (*n*)	27.6 (27)	25.0 (1)	ns
**CRP**, mg/L, mean ± SD	128.8 ± 93.2	93.1 ± 36.1	ns
>100 mg/L, % (*n*)	57.1 (56)	50 (2)	ns
**IL-6**, pg/mL, mean ± SD	283.2 ± 618.1	105.4 ± 119.9	ns
>100 pg/mL, % (*n*)	26.5 (26)	25.0 (1)	ns
**PCT**, ng/mL, mean ± SD	3.0 ± 11.9	0.3 ± 0.3	<0.01
>0.25 ng/mL, % (*n*)	48.0 (47)	25.0 (1)	ns
**d-dimer**, ng/mL, mean ± SD	4371.1 ± 14900	1550.2 ± 2423.9	ns
**Baseline WHO**, median (IQR)	4 (4–5)	4 (4–4)	ns
**Admission SpO_2_**, %	84.0 ± 13.8	83.5 ± 4.5	ns
**Lung involvement**, %	40.9 ± 25.8	53.7 ±26.9	ns
**Oxygen therapy**, % (*n*)	94.5 (93)	100.0 (4)	ns
**Mechanical ventilation**,% (*n*)	25.5 (25)	25.0 (1)	ns

ns—no significant difference (*p* > 0.05); ALC—absolute lymphocyte count, ANC—absolute neutrophil count, NLR—neutrophil-to-lymphocyte ratio, PLC—platelet count, WBC—white blood cells count, CRP—C-reactive protein, IL-6—interleukin-6, PCT—procalcitonin. *—leukemia patients (*n* = 7) were excluded from this statistic.

**Table 4:** The demographic, laboratory, and clinical characteristics of fully vaccinated patients (*n* = 723) who presented their first COVID-19 symptoms less and more than 180 days after receiving the last dose.

Parameter	≤180 Days (*n* = 361)	>180 Days (*n* = 362)	*p*-Value
**Age**			
Mean ± SD, years	63.6 ± 15.2	72.7 ± 14.9	<0.001
Range, years	18–93	19–99	
>70 years, % (*n*)	33.5 (121)	65.5 (237)	<0.001
Female/Male, % (*n*)	42.9 (155)/57.1 (206)	44.7 (162)/55.3 (200)	ns
**BMI**			
Mean ± SD, kg/m^2^	28.8 ± 5.8	28.3 ± 5.6	ns
Range, kg/m^2^	15.6–54.9	17.6–49.0	
Obesity (≥30.0), % (*n*)	28.5 (103)	24.6 (89)	ns
**Comorbidities**, % (*n*)	79.5 (287)	90.3 (327)	<0.001
Asthma, % (*n*)	6.9 (25)	8.3 (30)	ns
Cardiovascular, % (*n*)	69.0 (249)	78.7 (285)	<0.01
Cancer, % (*n*)	11.6 (42)	13.8 (50)	ns
Chronic kidney disease, % (*n*)	15.0 (54)	21.6 (78)	ns
Diabetes, % (*n*)	29.4 (106)	31.5 (114)	ns
**Immunosuppression**, % (*n*)	11.1 (40)	11.0 (40)	ns
**Humoral non-responders**, % (*n*)	12.4 (45)	13.5 (13.5)	ns
**ALC** *, ×10^3^/µL, mean ± SD	1.5 ± 4.9	1.6 ± 5.9	ns
**ANC**, ×10^3^/µL, mean ± SD	5.7 ± 3.3	6.2 ± 5.9	ns
**NLR** *, mean ± SD	7.4 ± 7.9	9.6 ± 5.2	ns
**PLC**, ×10^3^/µL, mean ± SD	215.9 ± 99.5	207.9 ± 99.4	ns
**WBC**, ×10^3^/µL, mean ± SD	8.4 ± 9.7	8.2 ± 8.7	ns
>11 × 10^3^/µL, % (*n*)	15.5 (56)	13.8 (50)	ns
**CRP**, mg/L, mean ± SD	90.3 ± 87.0	81.2 ± 74.8	ns
>100 mg/L, % (*n*)	37.2 (134)	31.0 (112)	ns
**IL-6**, pg/mL, mean ± SD	110.4 ± 405.4	108.0 ± 294.7	ns
>100 pg/mL, % (*n*)	13.3 (48)	13.5 (49)	ns
**PCT**, ng/mL, mean ± SD	3.5 ± 17.1	3.4 ± 16.8	ns
>0.25 ng/mL, % (*n*)	23.5 (85)	22.9 (83)	ns
**D-dimer**, ng/mL, mean ± SD	1504.4 ± 3472.7	2563.8 ± 9489.1	<0.05
**Hospital stay**, days	11.5 ± 7.7	12.1 ± 9.3	ns
**Baseline WHO**, median, (IQR)	4 (4–5)	4 (4–5)	ns
**Admission SpO_2_**, %	90.1 ± 7.3	89.9 ± 8.5	ns
**Lung involvement**, %	27.0 ± 21.5	28.4 ± 21.4	ns
**Oxygen therapy**, % (*n*)	79.5 (287)	82.9 (300)	ns
**Mechanical ventilation**, % (*n*)	4.7 (17)	3.3 (12)	ns

ns—no significant difference (*p* > 0.05); ALC—absolute lymphocyte count, ANC—absolute neutrophil count, NLR—neutrophil-to-lymphocyte ratio, PLC—platelet count, WBC—white blood cells count, CRP—C-reactive protein, IL-6—interleukin-6, PCT—procalcitonin. *—leukemia patients (*n* = 7) were excluded from this statistic.

## Data Availability

The data presented in this study are available from the corresponding author on reasonable request.
